# Relevant mechanisms of MAIT cells involved in the pathogenesis of periodontitis

**DOI:** 10.3389/fcimb.2023.1104932

**Published:** 2023-02-21

**Authors:** Xinrong Jiang, Qingtong Zhao, Zhanyu Huang, Fengyu Ma, Kexiao Chen, Zejian Li

**Affiliations:** ^1^ Hospital of Stomatology, The First Affiliated Hospital of Jinan University, Jinan University, Guangzhou, Guangzhou, Guangdong, China; ^2^ Department of Stomatology, The Sixth Affiliated Hospital of Jinan University, Dongguan, Guangdong, China; ^3^ College of Stomatology, Jinan University, Guangzhou, Guangdong, China; ^4^ Chaoshan Hospital, The First Affiliated Hospital of Jinan University, Chaozhou, Guangdong, China

**Keywords:** mucosal-associated invariant T cell, periodontitis, IL-17, alveolar bone resorption, immune reaction

## Abstract

Mucosal-associated invariant T (MAIT) cells are a group of unconventional T cells that are abundant in the human body, recognize microbial-derived vitamin B metabolites presented by MHC class I-related protein 1 (MR1), and rapidly produce proinflammatory cytokines, which are widely involved in the immune response to various infectious diseases. In the oral mucosa, MAIT cells tend to accumulate near the mucosal basal lamina and are more inclined to secrete IL-17 when activated. Periodontitis is a group of diseases that manifests mainly as inflammation of the gums and resorption of the alveolar bone due to periodontal tissue invasion by plaque bacteria on the dental surface. The course of periodontitis is often accompanied by a T-cell-mediated immune response. This paper discussed the pathogenesis of periodontitis and the potential contribution of MAIT cells to periodontitis.

## Introduction

Mucosal-associated invariant T (MAIT) cells are a type of unconventional T cell. They were first discovered in human peripheral blood in 1993 (Porcelli et al., 1993) and were named mucosal-associated invariant T cells in 2003 because of their tendency to accumulate in the mucous membranes of the lamina propria of the intestinal tract of humans and mice (Treiner et al., 2003). In peripheral blood, most MAIT cells are CD4^-^CD8^+^ and CD4^-^CD8^-^(double negative, DN), and a small portion of them are CD4^+^. MAIT cells express a semi-invariant T-cell antigen receptor (TCR) with a constant Vα7.2 fragment coupled with Jα33, Jα20, or Jα12 and paired with a limited Vβ chain (Vβ2/Vβ13) (Tilloy et al., 1999; Reantragoon et al., 2012; Lepore et al., 2014). The function of MAIT cells will also change with different TCR chains (Le Bourhis et al., 2010; Dias et al., 2017). MAIT cells can be found wherever traditional T cells exist, accounting for 1-10% of the total T cells (Koay et al., 2018; Nel et al., 2021), among which 20-40% are distributed in the liver, 3% in the intestinal mucosa, 3% in the lung, 1–3% in the stomach, 3% in the genital mucosa, 2% in the oral mucosa, and 2% in the skin (Hinks and Zhang, 2020; Legoux et al., 2020; Nel et al., 2021). The number and frequency of MAIT cells varies from person to person, and the level of MAIT cells is also associated with age: A very low number of MAIT cells can be observed in umbilical cord blood, which may be due to the extremely limited number of microorganisms present in the uterus. The number of MAIT cells gradually increases in the blood of newborns. It reaches its peak at 30~40 and then begins to decrease (Walker et al., 2012; Leeansyah et al., 2014; Novak et al., 2014). Although scientists discovered MAIT cells more than two decades ago, it is only in recent years that they have attracted attention and research. Current research on MAIT cells is conducted mainly in the fields of oncology, infectious diseases, and autoimmune diseases to study the changes and immune role of MAIT cells.

**Figure 1:**
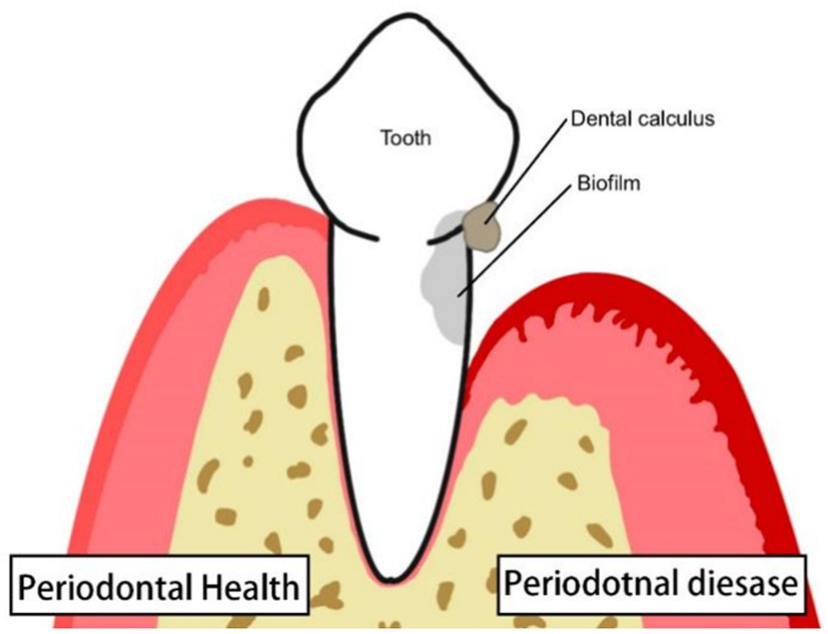
Healthy periodontal tissue and inflamed periodontal tissue: Periodontitis is characterized by hyperplasia of the gingival epithelium, gingival swelling, low gingival attachment, and alveolar bone atrophy.

Periodontitis is periodontal tissue inflammation caused by the bacterial biofilm covering the teeth. The main clinical manifestations include redness, swelling, bleeding, bad breath, tooth mobility, bleeding on probing (BOP), loss of attachment, and periodontal pocket formation. The pathological manifestations mainly included epithelial hyperplasia, loss of collagen fibres, and alveolar bone destruction and resorption (**Figure 1**). Early periodontitis may show no obvious symptoms, or only gingivitis symptoms, such as redness of the gums, bleeding when brushing teeth, and bad breath; therefore, patients often feel no discomfort. In later stages of periodontal disease, the periodontal tissue is absorbed due to inflammatory invasion, and the teeth appear to be loose and displaced. The teeth may even fall off when biting hard objects (Kinane et al., 2017). T cells play an essential role in the immune response to periodontitis. The proportion of T cells in patients with periodontitis is dysregulated, and various inflammatory cytokines are secreted in large quantities. Metabolic disorders occur in local tissues, gingival fibres are destroyed, alveolar bone is absorbed, and teeth lose periodontal tissue support and begin to loosen and fall off. In recent years, periodontal diseases have replaced dental caries and become the most important cause of tooth loss in adults (Ramseier et al., 2017; Forouzanfar et al., 2020). As a subclass of T cells, MAIT cells may also play an immune role in periodontal disease.

## Biological characteristics of MAIT cells

MAIT cells differentiate from the naive CD161^++^ CD8^+^ T-cell pool (Walker et al., 2012) and are initially present in the thymus. They then leave the thymus and move to peripheral tissues. Granzymes are usually expressed by circulating blood MAIT cells in the resting state, and granzymes A and K are the most common (Kurioka et al., 2015). When MAIT cells are activated, the expression of cytokines will be more abundant.

MAIT cells were first identified as MR1-restricted T cells, which require activation by MHC class-I related protein 1 (MR1) and play an immune role. Martin et al. found in animal experiments that MAIT cells were undetectable in germ-free mice and in mice born lacking MR1 (Martin et al., 2009). It has been suggested that microorganism sources of MR1 and antigen are required for the activation and differentiation of MAIT cells (Treiner et al., 2003; Martin et al., 2009; Corbett et al., 2014). Researchers later discovered MR1 ligands and concluded that MR1 cannot directly activate MAIT cells; it needs other ligands to do so (Kjer-Nielsen et al., 2012; McWilliam et al., 2015). The ligands that specifically bind to MR1 include vitamin B9 (folic acid)-derived 6-formylpterin (6-FP) and acetyl-6-formylpterin (Ac-6-FP), but the binding of 6-FP to MR1 does not activate MAIT cells (Kjer-Nielsen et al., 2012; Eckle et al., 2014). The ligands that couple specifically to MR1 and activate MAIT cells are vitamin B2 (riboflavin)-derived ribityllumazines, including 7-hydroxy-6-methyl-8-D-ribityllumazine (RL-6-ME-7-OH), 6,7-dimethyl-8-D-ribityllumazine (RL-6,7-DIME), and reduced 6-hydroxymethyl 8-D-ribityllumazine (RL-6-CH2OH) (Kjer-Nielsen et al., 2012; Eckle et al., 2014). The recent addition to the riboflavin-associated MR1 ligand family is an early intermediate in riboflavin synthesis, 5-amino-6-D-ribitylaminouracil (5-A-RU), which forms 5-(2-oxoethylideneamino)-6-D-ribitylaminouracil (5-OE-RU) and 5-(2-oxopropylideneamino)-6-D-ribitylaminouracil (5-OP-RU) by nonenzymatic reaction with small molecules of other metabolites (such as glyoxal or methylacetaldehyde). These are the most potent MAIT cell activators currently known (Corbett et al., 2014). MR1 quadruplets were also found to specifically detect mouse and human MAIT cells (Reantragoon et al., 2013; Birkinshaw et al., 2014; Rahimpour et al., 2015). This led to a deeper understanding of the physiology of MAIT cells, which was of great help in the research that followed.

There are two methods to activate MAIT cells. The more common method is dependent on the MR1 pathway: 5-OE-RU and 5-OP-RU produced by bacteria (such as *Mycobacterium, Escherichia coli, Lactobacillus, Staphylococcus, Shigella fowleri, Salmonella, Mycobacterium and Clostridium (* Le Bourhis et al., 2010; Le Bourhis et al., 2013)) or fungi (such as *Saccharomyces, Candida, and Aspergillus* (Le Bourhis et al., 2010; Dias et al., 2017; Jahreis et al., 2018; Meermeier et al., 2018)) are captured by MR1 to form antigens, which then promote the activation of MAIT cells (Treiner et al., 2003; Davanian et al., 2019). Activated MAIT cells start to secrete inflammatory response cytokines, kill pathogenic microorganisms and generate immune responses to bacterially infected cells (Provine and Klenerman, 2020), in addition to building a bridge between innate and adaptive immunity. Mammalian somatic cells lack the riboflavin synthesis pathway (Kjer-Nielsen et al., 2012). Therefore, the product of this pathway can be used as a molecular marker to test and confirm the occurrence of microbial infection in mammals.

If the pathogenic microorganisms that invade the body are bacteria or viruses that cannot synthesize riboflavin and do not have 5-OE-RU and 5-OP-RU that can be captured by MR1, then MAIT cells can be activated through a MR1-independent pathway. Following the invasion of microorganisms such as viruses that cannot synthesize riboflavin, dendritic cells in infected tissues are stimulated to produce large quantities of cytokines such as TNF-α, IL-18, IL-12, and IFN (Phetsouphanh et al., 2021; Provine et al., 2021). Through stimulation by these high levels of cytokines, MAIT cells can also be activated directly without TCR ligands (Hinks and Zhang, 2020; Provine and Klenerman, 2020) and are involved in the immune response. Many studies have shown that the high level of CD69 expression by activated MAIT cells is positively correlated with the high level of IL-18 in the blood (Haga et al., 2016; Law et al., 2019; Yang et al., 2022).

The two activation pathways mentioned above are not entirely separate. In some cases, the activation of MAIT cells depends on both MR1 and cytokines. Ussher et al. (2016) found that early MAIT cell activation was MR1-dependent rather than cytokine-dependent, and that late activation involved both mechanisms. After activation, the expression of the surface molecules CD25 and CD69, cytokines (e.g.,IFN-γ, IL-17, TNF-α and IL-22), intracellular perforin, and granzyme B is enhanced, mediating the apoptosis of infected cells, activating innate immune cells, and recruiting adaptive immune cells.

After pathogenic microorganisms invade the body, MAIT cells are activated by the two pathways mentioned above and rapidly migrate from the peripheral blood to the site of infection. The activated MAIT cells then begin to secrete a large number of proinflammatory cytokines, such as TNF-α, IFN-γ, and IL-17, and actively participate in the immune response, killing infected cells and maintaining immune homeostasis (Davanian et al., 2019).

Notably, different cytokines can stimulate MAIT cells to secrete various subclasses of granzymes, but their exact relationships and mechanisms of action remain less clear. Additionally, in infectious diseases, there are differences between viral and bacterial infections, and the same condition may present different disease courses (Rudak et al., 2018). In HIV patients, the number of MAIT cells increases sharply at first and then decreases as the disease progresses from early to late stages, but the number of MAIT cells is also reduced if the patient is undergoing antiviral therapy. The proportion of MAIT cell subsets varies with disease progression. According to follow-up records, the proportion of DN MAIT cells and the CD8^+^ MAIT cells decreased with disease progression. The proportion of MAIT cells decreases, but the total number of MAIT cells remains progressively lower (Phetsouphanh et al., 2021). Programmed death 1 (PD-1) and GrB were higher than their preinfection rate (Lal et al., 2020). The longer the course of the disease, the more irreversibly damaged the immune function of MAIT cells becomes; in addition, their immune response and function against other pathogens, such as Mycobacterium tuberculosis, are significantly reduced (Balfour et al., 2021).

## MAIT cells and oral diseases

Williams et al. (2021), through single-cell RNA sequencing (scRNA-seq) and immunohistochemical experiments, found MAIT cells residing in buccal mucosa and gums, primarily clustered between epithelial tissue and connective tissue. Sobkowiak et al. (2019) found that MAIT cells reside in the buccal mucosa, which, CD103^+^ unlike peripheral blood, mainly expressed CD4^+^, CD69^+^, and CD103^+^. CD103^+^ MAIT cells are more active when subjected to external stimulation, which may be the main force driving mucosal resident MAIT cells to produce IL-17. Kim et al. (2022) found that resident MAIT cells in gingival tissue tended to gather near the basement membrane of gingival tissue because chemokines CCL20 and CXCL16 were highly expressed in connective tissues near the basement membrane, while their receptors CCR6 and CXCR16 were highly expressed by gingival MAIT cells.

Apical periodontitis (AP) is a type of dental pulp disease caused by bacterial infection that can cause the destruction of gum tissue and bone resorption. Compared with healthy gingival tissue, CD4^+^ MAIT cells are more prevalent in AP tissues, and MAIT cells with TCR of Vα7.2Jα33 are dominant, followed by MAIT cells with TCR of Vα7.2Jα12 and Vα7.2Jα20 (Davanian et al., 2019). The levels of IL-17 and IFN-γ, and specially TNF-α, are significantly higher in AP tissue (Davanian et al., 2019). The level of riboflavin accumulation in AP tissues was higher than that in the normal group, and microbiome analysis of AP tissues revealed significant enrichment of riboflavin synthesis pathway genes (Davanian et al., 2019). It was proven that the microorganisms in AP induced the activation of MAIT cells through the synthesis of riboflavin, caused the infiltration of MAIT cells into diseased tissue and promoted the production of these cytokines.

Oral lichen planus (OLP) is an autoimmune chronic inflammatory disease that occurs mainly on the buccal mucosa but also on the gums. The occurrence of OLP may also be accompanied by *Candida albicans* infection (Mun et al., 2016). In the OLP group, the number of MAIT cells in OLP tissues was higher than that in circulating blood. Moreover, the number of CD4^+^, DN, CD69^+^ and CD103^+^ MAIT cells increased in the damaged tissues, and the number of CD69^+^ and CD103^+^ MAIT cells was positively correlated with the RAE score. The production of GrB and PD-1 by MAIT cells in OLP tissues was higher than that in the control group (Yang et al., 2022).

## MAIT cells and periodontitis

In healthy periodontal tissue, CD4^+^ T cells generally account for a higher proportion of T cells, followed by CD8^+^ T cells and γδ T cells (Dutzan et al., 2016). They inhibit the differentiation of osteoclasts by secreting anti-inflammatory factors, such as IL-10 and TGF-β, to achieve a stable dynamic balance between bone resorption and osteogenesis. While the oral flora is disordered and pathogenic microorganisms invade the periodontal tissue, dendritic cells, monocytes, macrophages, and Langerhans cells in the periodontal tissue function in antigen presentation, causing the activation and proliferation of antigen-specific T cells and aggregation at the inflammatory site. The proportion of CD4^+^/CD8^+^ cells decrease and produce a large number of inflammatory cytokines (such as IL-6, IL1β, TNF-α, etc.), which then induce the differentiation and proliferation of all kinds of Th cells and cause them to secrete TNF-α, IFN-γ, IL-17 and other cytokines (Figueredo et al., 2019). Thus, harmonious homeostasis is over.

MAIT cells, as T cells, are naturally involved in this immunity battle. *Porphyromonas gingivalis (P. gingivalis), Fusobacterium nucleatum (F. nucleatum), and Actinobacillus actinomycetemcomitans (A. actinomycetemcomitans)* are the most common causative agents of chronic periodontitis with riboflavin synthesis (Mombelli et al., 1994; Schenkein et al., 2000; Vitreschak et al., 2002; Loozen et al., 2014; Gutiérrez-Preciado et al., 2015; Brennan and Garrett, 2019). In the form of gum porphyrin monocytosis and nuclear bacteria, the level of riboflavin synthesis is higher than the level of the suspension state (Ali Mohammed et al., 2021). In addition, a large number of activated MAIT cells were transferred from peripheral blood, and the disease-producing cells were developed by unactivated MAIT cells and activated MAIT cells. The activation of MAIT cells significantly increased MAIT cell uptake, confirming MAIT cell activation, which induces osteoblast formation and bone resorption (Cho et al., 2020). This provides important clues to the pathogenesis of MAIT cells involved in periodontitis.

Kim et al. found that the absolute number of gingival MAIT cells was higher in patients with periodontitis than in healthy individuals. For a given patient with periodontitis, the percentage of MAIT cells in their gums was higher than the percentage of MAIT cells in their circulating blood. However, the difference in the proportion of MAIT cells among T cells in the gum tissues of the healthy and periodontitis populations was not statistically significant (Kim et al., 2022). Williams et al. detected an increase in the number of all types of immune cells in the gingival tissue of patients with periodontitis by scRNA-seq and flow cytometry. However, only plasma cells and neutrophils increased in proportion to disease, with no statistically significant change in the proportion of T cells (Williams et al., 2021). Perhaps we can deduce that although the number of MAIT cells is increased in the gums of periodontitis patients, the proportion of T cells is not much different from that of healthy people. In patients with periodontitis, the absolute number of MAIT cells in circulating blood decreased, while the proportion of CD69^+^ MAIT increased and the blood levels of IL-17 and TNF-α increased (Kim et al., 2022). The IL-17 expression of MAIT cells in gums was higher than that of MAIT cells in circulating blood (Kim et al., 2022). The number of cells expressing CCL20 and CXCL16 in the gum tissue of periodontitis patients also increased (Kim et al., 2022), demonstrating that MAIT cells can migrate to inflammatory tissue to participate in the immune response (Kim et al., 2022). Compared with peripheral blood, under the stimulation of pathogenic microorganisms or high levels of cytokines, oral mucosa MAIT cells highly expressed CD69, CD103, HLA-DR, and PD-1 and were more prone to produce IL-17 and TNF (Figueredo et al., 2019; Sobkowiak et al., 2019; Nel et al., 2021; Kim et al., 2022). This may indicate that MAIT cells are more active in the oral mucosa because it is easily exposed to microbial stimulation.

Some viruses can cause inflammation of periodontal tissues in addition to immune system disorders. The virus most often associated with periodontitis is HIV. The following periodontal lesions are internationally recognized as being associated with HIV infection: linear gingival erythema (LGE), necrotizing ulcerative gingivitis (NUG), and necrotizing ulcerative periodontitis (NUP). Scholars believe that there is a bidirectional association between HIV and periodontitis, with the gingiva serving as the site of HIV harbouring. This is because periodontal tissues highly express CCR5 and CXCR4, which are HIV-1 binding receptors, in patients with chronic periodontitis combined with HIV (Jotwani et al., 2004; Boy et al., 2009). When cells possess CD4 and CCR5 and/or CXCR4 receptors, HIV can fuse with the host cell membrane and thus complete the process of infecting the target cells. Some scholars have also demonstrated through clinical trials and return visits that patients who have only received anti-HIV therapy have a corresponding reduction in the severity of periodontitis (Valentine et al., 2016; Pólvora et al., 2018). The deeper the probing depth (PD) of the periodontal pocket and the lower the clinical attachment level (CAL) of the probing site, the higher the viral level in the gingival sulcus fluid of the patient (Maticic et al., 2000). From these studies and the literature, we can speculate that HIV can indeed promote the development of periodontitis and that deep periodontal pockets provide a reservoir for these viruses, creating a vicious cycle of periodontal tissue destruction.

Regarding the related mechanism of periodontal disease caused by these viruses, scholars believe it may be the symbiotic effect of bacteria and viruses (Slots and Slots, 2019; Chen et al., 2020). The invasive bacterial infection damages the periodontal tissue, and viruses invade the periodontal tissue during this period. Consequently, the immune response triggered by the virus during this period facilitates continued bacterial invasion. Naturally, the virus itself can also cause some direct damage to the periodontal tissue by eliciting a local immune response. In addition, some viruses can reduce the immunity of the host through immunosuppression (Graves et al., 2019) or immune escape (Jochum et al., 2012), resulting in local immune dysregulation of the periodontal tissue, promoting the secretion and release of inflammatory cytokines and reducing the host immune response (Chiang and Liu, 2018). After the viral invasion of periodontal tissues, immune cells are stimulated to fight back, with the high secretion of various interleukins, interferons, and tumour necrosis factors by dendritic cells and T cells, and under the stimulation of high levels of cytokines, MAIT cells are activated through the MR1-independent pathway and follow the relay to participate in the antiviral immune response (Flament et al., 2021) and start to secrete various cytokines, especially IL-18 and IL 17a (Parrot et al., 2020), which further facilitates the MR1-independent activation pathway of MAIT cells, producing IFN-γ and TNF-α, inhibiting viral replication, attacking infected cells (Loh et al., 2016), and mediating the development of periodontitis. IL-17 secreted by MAIT cells also stimulates the differentiation of Th17 cells, further exacerbating the inflammatory response.

IL-17 is a crucial cytokine associated with the development of periodontitis. IL-17 can upregulate RANKL expression and downregulate OPG expression through the NF-κB signalling pathway, resulting in changes in the RANKL/OPG ratio, indirectly promoting the differentiation of osteoblasts and participating in bone resorption. IL-17 also induces proinflammatory factors such as TNF-α, IL-1β and IL-6 in T cells, enhances the activity of protein hydrolases (neutrophil protease, peroxidase, matrix metalloproteinase, etc.), inhibits the secretion of anti-inflammatory cytokines, such as IL-10 and TGF-β, and accelerates the breakdown of collagen fibres in periodontal tissues (Dutzan et al., 2018). In addition, IL-17 can act directly on osteoclasts and directly participate in bone resorption while stimulating the differentiation of Th17 cells, further increasing the levels of cytokines such as IL-17 and IL-6, activating the STAT signalling pathway, inducing RORγt transcription factor expression, and exacerbating damage to the local area (Huang et al., 2021). It has also been shown that patients with periodontitis have abnormal bone metabolism in the alveolar bone, with some disturbances in Ca metabolism, which hinders alveolar bone production (Kuraji et al., 2021), thus shifting the dynamic balance towards bone resorption.

TNF-α is an important inflammatory factor that has a close relationship with nuclear factor κB (NF-κB) and can activate NF-κB *in vivo* by stimulating signals outside the cell, enhancing the gene transcription of TNF-α and indirectly promoting the expression of TNF-α. Subsequently, TNF-α activates NF-κB again through a positive feedback mechanism, which not only further increases the secretion of TNF-α but also induces. In addition to triggering immune effects and memory immune responses through NF-κB, TNF-α promotes MAIT cells to secrete more cytokines (Jiang et al., 2018) and increase the content of inflammatory factors. TNF-α also enhances the expression of RANKL, increases the activity and number of osteoclasts, induces immune cells to express chemotactic mediators, increases the production of matrix metalloproteinases (MMPs), destroys collagen fibres in gingival tissues, stimulates apoptosis of stromal cells, and limits the self-repair of periodontal tissues. Branetti et al. confirmed through clinical trials that the concentration of TNF-α in peripheral blood mononuclear cell culture supernatants of patients with periodontitis was only slightly higher than that of normal controls, but after lipopolysaccharide in experimental periodontitis, the TNF-α levels were significantly increased by lipopolysaccharide stimulation. In experimental periodontitis, TNF-α antagonists reduced bone loss by 50% (Oates et al., 2002).

**Figure 2:**
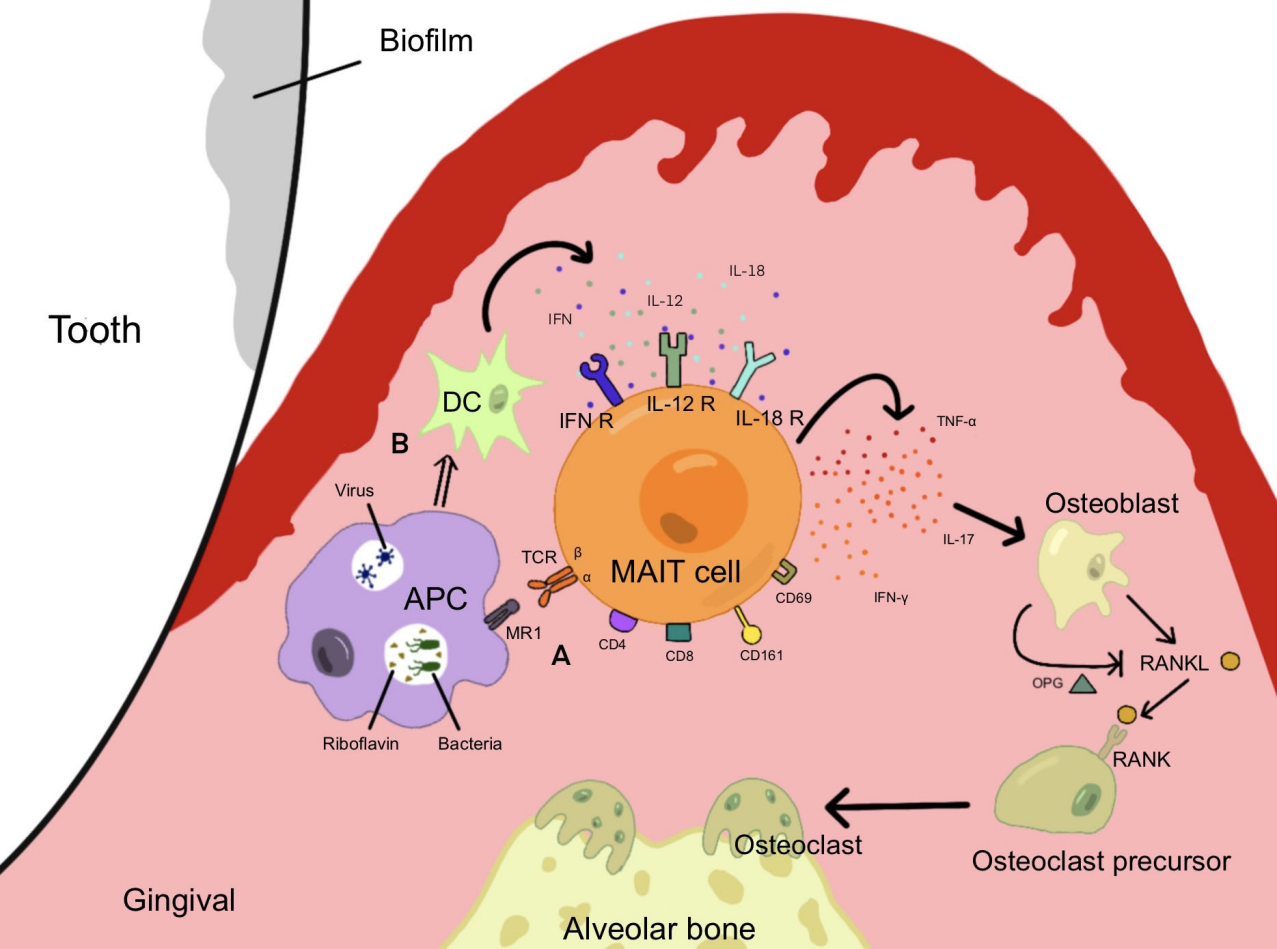
MAIT cells are activated and produce cytokines under different pathways, which induce osteoblasts to form osteoclasts and induce alveolar bone resorption, promoting the development of periodontal inflammation. **(A)** Pathogenic bacteria capable of synthesizing riboflavin invade periodontal tissue and activate MAIT cells through the MR1 pathway. **(B)** Viruses invade periodontal tissue and produce cytokines such as IL-12 and IFN through DCs, causing MAIT cells to be activated through the MR1-independent pathway.

In conclusion, IL-17 and TNF-α are closely related to periodontitis, and MAIT cells that can secrete these cytokines might affect the course of periodontitis. It is not difficult to conclude that when pathogenic microorganisms invade periodontal tissues, periodontitis pathogens secrete riboflavin at a high level on the subgingival plaque biofilm and activate MAIT cells. When the virus invaded periodontal tissue, it stimulated the secretion of cytokines such as IL-18, further promoting the activation of MAIT cells and the production of inflammatory cytokines such as IL-17 and TNF. Ca metabolism was disturbed, the RANKL pathway was upregulated, alveolar bone was absorbed, and periodontal tissue was destroyed (**Figure 2**).

## Conclusion

MAIT cells have been extensively researched in many infectious diseases and have been shown to have important immune functions in infectious diseases through a large amount of experimental and clinical data. The literature on MAIT cells in oral diseases is still scarce. In the case of existing research, we can conclude that there are resident MAIT cells in the buccal mucosa and gums, and after oral mucosa disease, circulating MAIT cells can be summoned to the affected tissue and be involved in the immune response. MAIT cells in the oral mucosa are more likely to express IL-17 and TNF after activation. These results show that MAIT cells are more or less involved in periodontitis immune responses. The role of MAIT cells in the onset and chronic course of periodontitis provides a new explanation for the pathogenesis of periodontitis, the core of which is the role of oral bacteria in activating MAIT cells. Further research is needed to assess whether MAIT cell activation actually increases the number of MAIT cells involved in periodontitis, whether MAIT cells are directly involved in the regulation of bone resorption and whether MAIT cells respond to common symbiotic oral microbes.

## Author contributions

XJ: Conceptualize and write the first draft; QZ: Review and revise manuscript;ZH: Revise manuscript; FM: Search for references; KC: Search for references; ZL: Review of manuscript.
